# Strength Use and Thriving at Work Among Chinese Nurses: The Mediating Roles of Control Beliefs About Stress and Cognitive Reappraisal

**DOI:** 10.1155/2024/5509059

**Published:** 2024-10-18

**Authors:** Baoyu Bai, Chengzhi Bai

**Affiliations:** ^1^Department of Psychology, Academy of Advanced Interdisciplinary Studies, Wuhan University, Wuhan, China; ^2^School of Philosophy, Wuhan University, Wuhan, China

**Keywords:** cognitive reappraisal, control beliefs about stress, nurse, strength use, thriving at work

## Abstract

**Aim:** This study aims to evaluate how strength use affects thriving at work among Chinese nurses, with a focus on the mediating effects of control beliefs about stress and cognitive reappraisal.

**Background:** Nurses' thriving at work is essential for their well-being, highlighting the importance of understanding factors that contribute to their thriving.

**Methods:** A comprehensive questionnaire was administered to measure nurses' strength use, control beliefs about stress, cognitive reappraisal, and thriving at work. Data were analyzed using SPSS 25.0, with mediation analyses conducted via the PROCESS macro. The study followed the STROBE checklist to ensure quality and transparency.

**Results:** Based on data from 434 nurses, strength use was positively related to thriving at work (*β* = 0.455, *p* < 0.001), control beliefs about stress (*β* = 0.375, *p* < 0.001), and cognitive reappraisal (*β* = 0.467, *p* < 0.001). Mediation analyses showed that both control beliefs about stress and cognitive reappraisal independently mediated the relationship between strength use and thriving at work, with indirect effects of 0.068 (95% CI [0.011, 0.141]) and 0.092 (95% CI [0.037, 0.154]), respectively. The serial mediation model was also significant (indirect effect = 0.067, 95% CI [0.025, 0.108]).

**Conclusions:** The study highlights the critical role of strength use in enhancing workplace thriving among Chinese nurses, with control beliefs about stress and cognitive reappraisal serving as key mediators.

**Implications for Nursing Management:** Encouraging strength use in healthcare and implementing programs to develop control beliefs about stress and cognitive reappraisal can enhance thriving at work, contributing to a more effective healthcare system.

## 1. Introduction

In positive organizational psychology, “thriving at work” is a key concept. It integrates individual vitality with continuous learning and skill development [1]. This principle emphasizes the essential synergy between personal energy and career advancement, serving as a key driver for creating a dynamic and enriching workplace atmosphere. Prem et al. [2] describe thriving at work as a state of well-being. It blends vitality—feeling alive, energized, and passionate about work—with continuous learning that fosters personal and professional growth. The central role of this state is evidenced by its links to improved job performance [3], innovation [4, 5], and reduced stress [3, 6], placing thriving at the heart of organizational effectiveness and employee health. The relevance of thriving extends to high-stress fields such as nursing, where its cultivation is not only beneficial but critical to maintaining the quality of health care and advancing the sector [7, 8]. Addressing the elements that enable or inhibit thriving in these environments is crucial and sets the stage for transformative practices in healthcare.

### 1.1. Strength Use and Thriving at Work

A novel method for promoting thriving at work involves the proactive use of employees' strengths. Strength use refers to the extent to which individuals apply their unique strengths and talents across various situations [9]. Rooted in positive psychology, this concept highlights focusing on what individuals do best to improve performance, engagement, and satisfaction at work [10]. Research by Bai et al. highlights the benefits of this approach for healthcare workers, demonstrating its potential to reduce depressive symptoms [11, 12], mitigate burnout [13, 14], increase job satisfaction [15], and promote posttraumatic growth [16].

Character strengths theory [17] provides a foundation for understanding the connection between strength use and thriving at work. According to this theory, when individuals perform tasks that align with their innate abilities, they experience greater competence, energy, and work engagement [17]. This enhanced engagement, in turn, fosters a sense of vitality, which is essential for thriving at work [2]. Based on this theoretical framework, we hypothesize that strength use will be positively associated with thriving at work (Hypothesis 1). The theory suggests that by leveraging their strengths, nurses can increase their engagement and vitality, leading to higher levels of thriving. Empirical research supports this theoretical framework. For example, studies have shown that strength use is a significant positive predictor of thriving at work [18, 19]. Longitudinal research further supports this positive relationship [20].

Although there is a growing body of research on strength use, most studies have focused on broad, generalized contexts and have not specifically addressed its application in nursing settings [21, 22]. Recent studies have begun to explore the nuances of strength use in specific professions, including healthcare (e.g., [11, 13]). However, the direct connection between strength use in nursing roles and thriving at work remains underexplored. This gap is particularly significant given the increased stress levels and burnout risks among healthcare professionals, as highlighted by studies conducted during and after the COVID-19 pandemic [23, 24]. Moreover, the cognitive and emotional mechanisms through which strength use influences thriving at work are not well understood. These gaps in the literature underscore the need for targeted research that examines how strength use can enhance job satisfaction and professional development among nurses. Addressing these gaps is crucial for refining theoretical frameworks on strength use and thriving at work and for developing effective interventions to foster a thriving workforce, particularly in high-stress professions like nursing.

Therefore, our research aims to address these academic gaps by examining how strength use affects thriving at work, with a particular focus on the mediating effects of control beliefs about stress and cognitive reappraisal. We intend to highlight the critical importance of strategically using personal strengths in nursing to enhance nurses' experiences of thriving at work. Based on the above theoretical and empirical considerations, we propose the following hypothesis:

**Hypothesis 1 .**

Strength use can be positively associated with nurses' thriving at work.

### 1.2. Strength Use, Control Beliefs About Stress, and Thriving at Work

We propose that control beliefs about stress may serve as a mediating factor in the relationship between strength use and thriving at work. Laferton, Stenzel, and Fischer [25] define control beliefs about stress as the belief in one's ability to effectively cope with stressors. This belief is critical in determining how individuals face challenges and manage demands in their work environments [26–28].

Lyubomirsky and Layous' [29] positive activity model suggests that engaging in positive activities, such as using strengths, enhances well-being by promoting positive thoughts, emotions, and behaviors. This model helps explain how strength use can lead to improved control beliefs about stress and, subsequently, enhance thriving at work. Strength use involves recognizing and applying personal strengths in daily tasks, which can increase an individual's sense of efficacy and control over their work environment [30]. Control beliefs about stress refer to beliefs in one's ability to effectively manage and mitigate stressors [25]. When individuals use their strengths, they are more likely to experience success and positive outcomes, which strengthens their belief in their ability to manage stress. This increased perception of control over stress contributes to their overall well-being and ability to thrive at work.

In support of this, empirical research across the fields of positive and occupational health psychology has consistently shown that the use of personal strengths is correlated with improved perceptions of control and efficacy [21, 30, 31]. Specifically, Bai et al. [14] provided evidence of this dynamic among Chinese nurses, noting a positive relationship between strength use and control beliefs about stress. Furthermore, the perceived ability to manage workplace stress has been associated with increased resilience and engagement [32], which are essential components of thriving in professional settings [3, 33].

This is particularly relevant in demanding healthcare settings, where a strong belief in the controllability of stress can significantly impact job satisfaction and personal development [34, 35]. Therefore, based on these theoretical insights and empirical findings, we propose the following hypothesis:

**Hypothesis 2 .**

Control beliefs about stress mediate the relationship between strength use and thriving at work.

### 1.3. Strength Use, Cognitive Reappraisal, and Thriving at Work

We also propose cognitive reappraisal as a mediator in the association of strength use with thriving at work. This key aspect of emotional regulation involves reinterpreting situations to change their emotional impact [36]. It helps individuals manage emotional responses to stressors, especially in challenging work environments [37]. The link between strength use, cognitive reappraisal, and thriving at work is supported by Fredrickson's Broaden-and-Build Theory [38]. This theory suggests that positive emotions broaden one's cognitive scope, promoting the development of personal resources. Strength use in the workplace can generate positive emotions [21, 30] and enhance cognitive reappraisal [37]. This leads to broader perspectives and more adaptive responses to work challenges, thereby fostering an individual's ability to thrive at work.

Empirical evidence supports the relationship between strength use and improved cognitive reappraisal skills. For example, Tang, Lyu, and Xu [39] found that individuals who regularly use their personal strengths in the workplace report higher levels of cognitive reappraisal. This is because using strengths such as analytical skills or creativity leads to positive emotional experiences, which in turn enhance one's ability to reinterpret challenges positively [17, 38]. Consider a nurse who uses her strength of empathy in her interactions with patients. By using empathy, she can reframe a challenging encounter with a patient as an opportunity to provide exceptional care. This positive reinterpretation not only reduces her emotional distress but also contributes to a thriving work environment by fostering a sense of professional fulfillment and resilience [40]. Thus, the ability to effectively use personal strengths enables individuals to better manage their emotional responses to workplace stressors, promoting greater well-being and thriving at work [41].

Furthermore, cognitive reappraisal, as an effective emotion regulation strategy, positively impacts thriving at work [42]. It enables individuals to alter their emotional responses to workplace stressors, fostering resilience and engagement, which are essential for thriving in demanding roles like nursing [37, 41]. Based on these theoretical foundations and potential empirical connections, we propose the following hypothesis:

**Hypothesis 3 .**

Cognitive reappraisal mediates the relationship between strength use and thriving at work.

### 1.4. Control Beliefs About Stress and Cognitive Reappraisal

Our study extends the hypothesis that control beliefs about stress can support cognitive reappraisal at work. Drawing on Lazarus and Folkman's [43] stress theory, individuals with strong control beliefs about stress are likely to engage in positive coping mechanisms such as cognitive reappraisal. They tend to view stress as manageable, which promotes constructive reinterpretation of stressors and increases the use of cognitive reappraisal to alter perceptions and emotional responses to stress.

Empirical studies support the link between control beliefs about stress and effective emotion regulation. For example, research by Deplancke et al. [44] demonstrated that individuals with robust stress management beliefs exhibit superior cognitive appraisal skills. These skills are critical to emotional intelligence and resilience, as they promote an individual's ability to adaptively manage emotions in the face of workplace challenges. Similarly, studies have shown that self-efficacy, which is closely related to stress management beliefs, is significantly associated with better cognitive appraisal skills [45, 46]. This relationship underscores the importance of control beliefs about stress in developing cognitive reappraisal skills, which are essential for emotional adaptability.

Ma et al. [42] found that employees with strong control beliefs about stress are more likely to use cognitive reappraisal, which enhances their well-being and job satisfaction. Furthermore, resilience training that strengthens control beliefs about stress also improves cognitive reappraisal, leading to better stress management and greater work engagement [32].

**Figure 1:**
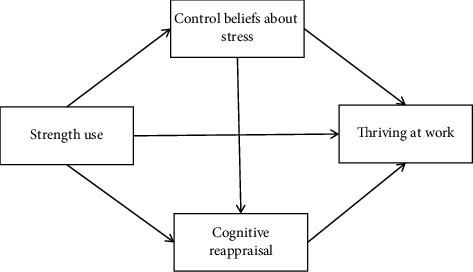
Theoretical framework of the study.

Our theoretical framework, as illustrated in Figure 1, hypothesizes that strength use positively influences control beliefs about stress, which in turn enhances cognitive reappraisal abilities, ultimately leading to higher levels of thriving at work.

Thus, informed by these theoretical insights and empirical findings, we advance the following hypothesis:

**Hypothesis 4 .**

Control beliefs about stress and cognitive reappraisal serially mediate the link between strength use and thriving at work.

## 2. Methods

### 2.1. Study Design

Our study was designed as a cross-sectional study. The cross-sectional design was chosen for its ability to capture and analyze data from a large sample at a single point in time, making it useful for exploring associations between variables such as strength use, control beliefs about stress, cognitive reappraisal, and thriving at work. Given the exploratory nature of the study, focusing on identifying relationships rather than causality, this design was appropriate. While it provides valuable insights into the current state of these variables, it is limited in its ability to infer causality, which should be considered when interpreting the findings.

### 2.2. Participants and Data Collection

This study was conducted in a hospital in Xiantao City, Hubei Province, China, in September 2023. Data were collected through an online survey distributed via WeChat groups using the Wenjuanxing platform, a widely used online survey tool in China. The research team contacted healthcare administrators to explain the purpose of the study and obtain permission to distribute the survey. Upon approval, the administrators shared the survey link with their nursing staff across multiple WeChat groups. The Wenjuanxing platform ensured anonymity and confidentiality of responses. Participants were informed of the voluntary nature of the study and that their responses would be used for research purposes only. To encourage participation, reminder messages were sent periodically through the same WeChat groups.

#### 2.2.1. Inclusion Criteria

Only registered nurses currently employed at the hospital were eligible to participate. Participants needed to have at least 1 year of work experience in their current role. Nurses who were actively working during the study period and provided informed consent were included in the study.

#### 2.2.2. Exclusion Criteria

Intern nurses or those still in training were excluded from participation. Additionally, nurses who were on leave (e.g., maternity leave and sick leave) during the data collection period were not eligible. Any participants who did not fully complete the survey or completed the survey in less than 100 s were excluded to ensure data quality.

### 2.3. Measures

All the measures used in this study have been validated in previous research and demonstrated strong reliability in similar contexts, particularly within the Chinese cultural setting.

#### 2.3.1. Strength Use at Work

In our research, we assessed employees' use of strengths using a subset of five items from the Strengths Use and Deficit Correction (SUDCO) questionnaire developed by van Woerkom et al. [47]. Examples of statements include “I seek opportunities to do my work in a way that best uses my strengths.” Participants rated their agreement with each item on a five-point Likert scale, ranging from 1 (*strongly disagree*) to 5 (*strongly agree*). High mean scores reflect high levels of strength use. The revised Chinese version of SUDCO has demonstrated strong structural validity and reliability [20]. In our study, the scale demonstrated excellent internal consistency as evidenced by a high Cronbach's alpha of 0.964.

#### 2.3.2. Control Beliefs About Stress

This measure was assessed using the three-item subscale of the Beliefs about Stress Scale by Laferton, Stenzel, and Fischer [25]. Each item is rated on a seven-point scale, ranging from 1 (*strongly disagree*) to 7 (*strongly agree*). The mean score of the three items was calculated to represent overall beliefs about stress. The reliability and validity of the scale in a Chinese context have been previously confirmed [26, 48]. In our research, the subscale showed a Cronbach's alpha of 0.826.

#### 2.3.3. Cognitive Reappraisal

To assess cognitive reappraisal, we used the Chinese version of the Emotion Regulation Questionnaire (ERQ), which was specifically designed to assess cognitive reappraisal by Zhu et al. [49] and originally developed by Gross and John [46]. The ERQ consists of two components, cognitive reappraisal and expressive suppression, and includes a total of 10 items (6 for cognitive reappraisal and 4 for expressive suppression). We used only the cognitive reappraisal subscale of the ERQ. Responses are scored on a seven-point scale, with higher mean scores indicating more frequent use of that strategy. The mean score of the six cognitive reappraisal items was calculated to represent the overall level of cognitive reappraisal. Previous research has shown that the ERQ has high reliability and validity in the Chinese cultural context [27]. The cognitive reappraisal dimension in this study had a Cronbach's alpha coefficient of 0.940, indicating strong internal consistency and reliability.

#### 2.3.4. Thriving at Work

In our study, we used Porath et al.'s [50] Thriving at Work Scale to measure thriving at work among Chinese nurses, focusing on vitality and learning. This 10-item scale, equally divided to assess feelings of energy and professional growth, includes statements such as “I feel energized at work” and “I often find myself learning.” Participants rated each item on a five-point Likert scale from 1 (*strongly disagree*) to 5 (*strongly agree*). The mean score of the 10 items was calculated to represent the overall level of thriving at work. Previous research has confirmed that the scale has high reliability and validity in the Chinese context [20]. In our study, it demonstrated excellent internal consistency, as evidenced by a Cronbach's alpha of 0.806, confirming its reliability in assessing thriving at work in the Chinese healthcare context.

#### 2.3.5. Sociodemographic and Organizational Variables

In addition to the primary variables of interest, the study collected several sociodemographic and organizational variables to account for potential confounding factors. These variables included gender (male and female), age group (20–29, 30–39, 40–49, and ≥50), education level (high school, associate degree, bachelor's degree, and postgraduate), marital status (single, married, divorced, and widowed), and professional title (junior nurse, senior nurse, and head nurse). These variables were included to better understand their influence on the study's primary outcomes.

### 2.4. Data Analysis Approach

Data analysis was conducted using SPSS25.0 and the PROCESS Macro for SPSS [51]. Specifically, we used Model 6 in the PROCESS Macro to test the hypothesized serial mediation model. Model 6 allows us to examine the indirect effects of strength use on thriving at work through two mediators: control beliefs about stress and cognitive reappraisal. This model is particularly appropriate for our study because it allows for the testing of sequential mediation effects, providing a comprehensive understanding of the underlying mechanisms.

To account for potential confounding variables, we included gender and age as control variables in our analysis. These control variables were selected based on their theoretical relevance and previous research indicating their potential influence on the outcome variables [52, 53]. Our approach included the use of 5000 bootstrap samples and 95% confidence intervals (CIs). This bootstrap method is highly regarded for its effectiveness in determining the significance of mediation effects, a methodology supported by Bolin [51].

### 2.5. Ethical Approval

The ethics committee of the Department of Psychology within the School of Philosophy at Wuhan University granted approval for this study (Approval No. 2021061601). Before participating, individuals were thoroughly briefed about the study's objectives, benefits, potential risks, and data usage. We ensured that each participant provided informed consent, acknowledging their comprehensive understanding and agreement to the study's terms and conditions.

## 3. Results

### 3.1. Statistical Overview and Correlation Assessment

A total of 437 questionnaires were initially collected from a sample of approximately 900 nurses. The response rate was approximately 48.6%. To ensure data quality, all questions were set as mandatory on the Wenjuanxing platform to prevent missing data. Additionally, we applied a filtering criterion based on response time: questionnaires completed in less than 100 s were excluded, as these were deemed to have been completed halfheartedly. This led to the exclusion of 3 questionnaires, resulting in a final sample of 434 valid responses used for analysis.  Table 1 provides the demographic information of the study participants.

A significant positive correlation was found among the variables of strength use, thriving at work, control beliefs about stress, and cognitive reappraisal, with all *p* values being less than 0.001 (see Table 2). The mean scores for these variables were as follows: strength use at work was 4.45 with a standard deviation (SD) of 0.74, control beliefs about stress 5.05 with an SD of 1.11, cognitive reappraisal was 5.26 with an SD of 1.07, and thriving at work was 3.72 with an SD of 0.56.

### 3.2. Analysis of Multiple Mediation Effects

We conducted a multiple mediation analysis using the PROCESS macro in SPSS 25.0 to examine whether control beliefs about stress and cognitive reappraisal concurrently mediate the relationship between strength use and thriving at work. The findings reveal that the use of strengths accounts for 21.30% of the variation in nurses' ability to thrive at work, supporting Hypothesis 1. Furthermore, the all-encompassing model sheds light on 39.20% of this variation. The findings highlighted the pivotal role of strength use as a considerable forecaster, contributing a significant percentage to the variance in workplace significant (*β* = 0.354, *p* < 0.001). Even after adjusting for the mediating factors, the influence of strength use on thriving at work remained direct and substantial (*β* = 0.180, *p* < 0.001), indicating a partial mediation by control beliefs about stress and cognitive reappraisal.

**Figure 2:**
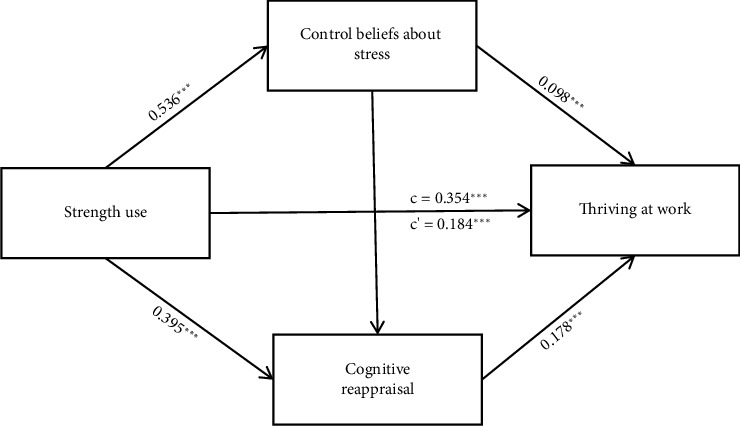
The mediating effects of control beliefs about stress and cognitive reappraisal between stress and thriving at work. Note: ⁣^∗∗∗^*p* < 0.001. Path coefficients are unstandardized.

The bootstrap method, shown in Table 3, confirmed the significance of the mediating roles of both control beliefs about stress (95% CI = [0.011, 0.141]) and cognitive reappraisal (95% CI = [0.037, 0.154]), supporting Hypotheses 2 and 3. The serial mediation model, leading from strength use to thriving at work via control beliefs about stress and cognitive reappraisal, was statistically significant (95% CI = [0.025, 0.108]), substantiating Hypothesis 4 (see Figure 2).

Lastly, the indirect effect comparison, as detailed in Table 3, indicated no marked difference in the mediating efficacy of control beliefs about stress and cognitive reappraisal in the strength use–thriving at work link (95% CI = [−0.130, 0.092]), suggesting their equivalent influence in this context.

## 4. Discussion

This study explored how strength use influences thriving at work among Chinese nurses, focusing on the mediating roles of control beliefs about stress and cognitive reappraisal. We found that both control beliefs about stress and cognitive reappraisal serve as independent mediators in the relationship between strength use and thriving at work. Our research also reveals a serial mediation model in which strength use predicts thriving at work through the sequential mediation of control beliefs about stress and cognitive reappraisal.

Our correlational analysis supports the positive relationship between strength use and thriving at work among Chinese nurses, aligning with previous studies that have documented strong associations between these variables (e.g., [3, 19]). These findings further underscore the crucial role of strength use in promoting thriving at work. However, it is important to consider how the cultural context of Chinese workplaces may influence this relationship. In collectivist cultures like China, individual strengths are often viewed as resources that contribute to collective outcomes, which may enhance the impact of strength use on thriving at work [54]. Nonetheless, the hierarchical structure common in Chinese organizations could either facilitate or hinder the use of personal strengths, depending on factors such as leadership style and organizational culture [55]. This dual influence suggests that while the general relationship between strength use and thriving at work may hold, the mechanisms through which this relationship operates could vary significantly across different cultural and organizational contexts. Future research should further explore these dynamics to better understand how strength use can be effectively leveraged to promote thriving in diverse work environments.

Promoting thriving at work through strength use can potentially lead to greater workforce stability and resilience [21], a critical consideration during public health emergencies. Overall, these findings provide valuable insights for healthcare organizations seeking to foster supportive and thriving work environments. By prioritizing and facilitating the use of strengths among their employees, healthcare leaders can significantly improve their workforce's ability to adapt, grow, and excel, even in challenging situations.

One of the key findings of our study was the role of control beliefs about stress and cognitive reappraisal as critical mediators in the relationship between strength use and thriving at work among Chinese nurses. This supports the positive activity model [29] and extends its application to healthcare. Our results indicate that engaging in strength use significantly enhances control beliefs about stress, thereby increasing nurses' experience of thriving at work. This finding aligns with previous research, which emphasizes that strong control beliefs about stress improve stress management and psychological outcomes [26, 27]. Bandura [45] similarly highlighted the role of self-efficacy, which is closely related to control beliefs about stress, in effective stress coping. However, occupational stress levels in nursing may modulate how these mediators function. In high-pressure environments like healthcare, the reinforcement of control beliefs about stress through strength use could have a more pronounced impact [56], suggesting that future research should explore these dynamics across different professional settings and cultural contexts. Integrating strength use into both professional and personal domains significantly enhances nurses' ability to manage stress and positively reframe challenging situations. This process not only strengthens control beliefs about stress but also fosters the resilience and adaptability needed to thrive in demanding healthcare environments [32].

Furthermore, we found that regular strength use is essential for developing cognitive reappraisal skills. Nurses who consistently apply their strengths become more adept at positively and constructively reframing stressful situations [13, 16, 39]. This cognitive shift not only reduces the emotional impact of stress but also fosters a more positive and proactive approach to the work environment [37]. As these cognitive reappraisal skills improve, nurses experience higher levels of thriving at work, characterized by greater professional fulfillment, engagement, and resilience [42].

These findings align with previous studies that have demonstrated the role of cognitive reappraisal in enhancing job satisfaction and reducing burnout, particularly in high-stress environments [57, 58]. However, while much of the existing literature has focused on cognitive reappraisal as a general stress management strategy, our study adds to the field by specifically linking regular strength use with the development of these skills in the nursing profession. This nuanced relationship suggests that strength use may serve as a crucial lever for promoting cognitive reappraisal, particularly in professions where emotional regulation is critical to maintaining well-being. Future research should continue to explore this linkage across different cultural and organizational contexts to determine the broader applicability of these findings. Such dynamics lay the foundation for a thriving professional life, marked by deep satisfaction and an enhanced capacity to grow and adapt within the complex field of nursing.

In addition, our research underscores that the mediating influence of control beliefs about stress is as significant as that of cognitive reappraisal in linking strength use to thriving at work. This finding highlights the equal importance of control beliefs about stress and cognitive reappraisal in bridging the gap between strength use and promoting thriving among healthcare professionals in China. Taken together, these findings shed light on the intricate relationship between strength use, thriving at work, and the psychological mechanisms underlying stress management and cognitive adaptation, providing valuable insights for fostering a resilient and satisfied healthcare workforce.

Interestingly, our final model demonstrated that strength use positively influences thriving at work through the serial mediation of control beliefs about stress and cognitive reappraisal. This suggests that nurses who engage more in strength use develop stronger control beliefs about stress, which in turn enhances their cognitive reappraisal, leading to greater thriving at work. Our findings align with existing literature, supporting the notion that individuals who frequently leverage their strengths foster more pronounced control beliefs about stress [16]. Deplancke et al. observed that enhanced control beliefs about stress are closely linked with more adept cognitive reappraisal techniques [44]. Furthermore, Ma et al. [42] identify cognitive reappraisal as a critical factor for thriving in professional settings.

This sequential interplay highlights the importance of nurturing personal strengths to develop psychological resilience and adaptive coping mechanisms. Strengthening control beliefs about stress allows individuals to better manage stressors and use cognitive reappraisal to positively reinterpret challenges [32, 42]. This process helps nurses achieve optimal functioning and well-being in their roles. These findings have practical implications for healthcare organizations. Implementing programs that promote strength use and resiliency training can enhance nurses' control beliefs about stress and cognitive appraisal skills, leading to improved well-being and job performance. Understanding these dynamics clarifies the relationship between strength use, thriving at work, and the psychological mechanisms underlying stress management and cognitive adaptation. Future research should explore these pathways in various contexts to validate and extend our findings.

### 4.1. Limitations and Future Directions

This study has several limitations that should be acknowledged, and these limitations have direct implications for interpreting our results. First, the study was conducted in a single hospital in China, which may limit the generalizability of the results to other settings and populations. The unique cultural and organizational context of this hospital could influence the relationships observed between strength use, control beliefs about stress, cognitive reappraisal, and thriving at work. Future research should include multiple hospitals and diverse geographic locations to assess whether these findings hold across different contexts.

Second, the cross-sectional design of this study limits our ability to make causal inferences about the relationships among the key variables. Although we found significant associations between strength use, control beliefs about stress, cognitive reappraisal, and thriving at work, longitudinal studies are needed to confirm these temporal relationships and determine causality.

Third, the use of self-report measures and the online survey distribution via WeChat may introduce biases that affect the validity of our findings. The online nature of the survey could have led to selection bias, as it may have predominantly reached more technologically savvy individuals. Additionally, response bias might have occurred, with participants providing socially desirable answers rather than accurate reflections of their experiences. These potential biases should be considered when interpreting the strength of the observed relationships.

Fourth, the study's use of categorical age data (20–29, 30–39, 40–49, ≥50) instead of continuous data limits the precision of age-related analyses. This could affect the detailed understanding of how age might interact with strength use, control beliefs about stress, and cognitive reappraisal. Future studies should collect age as a continuous variable to allow for more nuanced statistical evaluations.

Finally, while our study highlights the importance of control beliefs about stress and cognitive reappraisal as mediators, it did not examine other potential mediators and moderators, such as organizational support and job demands, which could also play significant roles in thriving at work. Future research should explore these additional factors to provide a more comprehensive understanding of the mechanisms underlying thriving at work. Furthermore, investigating the effectiveness of interventions aimed at enhancing strength use, control beliefs about stress, and cognitive reappraisal could provide valuable insights into their long-term impacts, especially in high-stress environments like healthcare.

## 5. Conclusion

Individuals who use their strengths at work not only thrive more but also enhance this effect through strong control beliefs about stress and cognitive reappraisal. These mediators play crucial roles in translating the use of strengths into tangible gains in thriving at work. Our findings reveal a serial mediation model, where control beliefs about stress precede and enhance cognitive reappraisal, further amplifying the positive impact of strength use on thriving at work.

While these findings provide a valuable framework for enhancing nurse well-being and effectiveness, future research should focus on exploring these dynamics in various cultural and organizational contexts to validate and extend these results. Longitudinal studies and mixed-method approaches could offer deeper insights into the causal relationships and underlying mechanisms. By continuing to investigate these areas, future research can further contribute to the development of interventions that promote thriving in high-pressure work environments like healthcare.

## 6. Implications for Nursing Management

The results of our study have profound implications for healthcare organizations and their leadership, particularly regarding the promotion of nurses' well-being and effectiveness. To translate these findings into actionable strategies, we propose the following recommendations:

### 6.1. Strength-Based Development Programs

Building on the strength-based development approach, healthcare organizations should implement a structured, multiphase intervention program similar to the integrative model proposed by Miglianico et al. [21]. This program would include

● Preparation and Commitment: Educating nurses about the strengths-based approach and its benefits. This phase ensures that nurses understand the process and are actively engaged from the start.
● Identification: Utilizing psychometric tools like StrengthsFinder or VIA-Survey, along with peer feedback, to help nurses identify their unique strengths.
● Integration: Supporting nurses in integrating their strengths into their professional identity, through exercises linking strengths to past successes and self-reflection activities.
● Action: Encouraging nurses to apply their strengths to specific job tasks, team projects, or organizational goals, with ongoing support and monitoring by supervisors or coaches.
● Evaluation: Regularly assessing the impact of strength application on job performance, well-being, and job satisfaction to ensure continuous improvement.

This comprehensive approach not only enhances nurse satisfaction and engagement but also improves the quality of patient care and fosters team cohesion [30].

### 6.2. Enhanced Stress Management Resources

Our findings emphasize the need for targeted education on managing work-related stress through strengthening control beliefs about stress. Healthcare organizations should consider introducing resilience training that incorporates cognitive-behavioral techniques tailored to the healthcare setting. For instance, regular training sessions could be offered that focus on cognitive restructuring, where nurses learn to reframe their thoughts about stressful situations. This could be complemented by a stress management toolkit, accessible online, featuring self-paced exercises and resources such as guided relaxation techniques, which nurses can use during breaks or after shifts. These efforts can empower nurses to feel more in control of stressors, ultimately leading to better coping strategies and reduced burnout [59].

### 6.3. Integration of Emotional Intelligence and Mindfulness Training

Given the mediating role of cognitive reappraisal in our study, we recommend incorporating emotional intelligence and mindfulness training into the core professional development curriculum for nurses. For example, mindfulness-based stress reduction programs could be offered as part of continuing education, focusing on developing nurses' abilities to remain calm and focused in high-pressure situations. Similarly, emotional intelligence training that includes modules on recognizing and managing emotions can be integrated into simulation-based learning sessions, where nurses practice reappraisal techniques in realistic scenarios. This approach not only fosters resilience but also enhances the overall work environment by promoting a culture of emotional awareness and support [46].

By linking these practical strategies directly to our study's findings, healthcare organizations can make informed decisions that will significantly improve nurse well-being, job satisfaction, and operational efficiency. Investing in these areas will not only support the personal and professional growth of nurses but also contribute to the delivery of higher-quality patient care and the overall effectiveness of healthcare systems.

## Figures and Tables

**Table 1:** Characteristics of the study sample.

	**Number**	**%**
Gender		
Female	413	95.2
Male	21	4.8
Age		
20–29	105	24.2
30–39	249	57.4
40–49	61	14.1
≥50	19	4.4
Education		
Technical secondary school	7	6.6
Junior college	70	43.4
Undergraduate	355	44.2
Postgraduate and above	2	5.9
Marital status		
Single	78	18.0
Married	350	80.6
Divorced	6	1.4
Profession title		
Nurse	39	9.0
Senior nurse	137	31.6
Supervisor nurse	237	54.6
Associate professor of nursing	21	4.8

*Note:* Nurse: Entry-level position, typically responsible for basic patient care and routine tasks under supervision. Senior Nurse: More experienced than entry-level nurses, often taking on additional responsibilities, leadership roles, and specialized tasks. Supervisor Nurse: Holds a supervisory role, overseeing the work of other nurses, managing nursing teams, and ensuring high standards of patient care. Associate Professor of Nursing: An academic position, typically involving teaching, research, and mentorship roles in a nursing education program, along with clinical practice responsibilities.

**Table 2:** Descriptive statistics and correlations among the key variables.

**Variables**	**M**	**SD**	**Correlation**			
			**1**	**2**	**3**	**4**
(1) Strength use at work	4.45	0.74	—			
(2) Control beliefs about stress	5.05	1.11	0.372⁣^∗∗∗^	—		
(3) Cognitive reappraisal	5.26	1.07	0.467⁣^∗∗∗^	0.626⁣^∗∗∗^	—	
(4) Thriving at work	3.72	0.56	0.455⁣^∗∗∗^	0.482⁣^∗∗∗^	0.561⁣^∗∗∗^	—

⁣^∗∗∗^*p* < 0.001.

**Table 3:** Mediation analysis results.

**Model pathways**	**Effect**	**Boot SE**	**95% CI**	
			**Lower**	**Upper**
Direct effect (Unstandardized)				
Strength use ⟶ thriving at work	0.184^a^	0.033	0.118	0.249
Standardized indirect effect				
Strength use ⟶ thriving at work	0.223^a^	0.034	0.157	0.289
Strength use ⟶ CBAS ⟶ thriving at work	0.068^a^	0.034	0.011	0.141
Strength use ⟶ CR ⟶ thriving at work	0.092^a^	0.030	0.037	0.154
Strength use ⟶ CBAS ⟶ CR ⟶ thriving at work	0.063^a^	0.022	0.025	0.108
IndEff (CBAS) minus IndEff (CR)	−0.023	0.057	−0.130	0.092

Abbreviations: CBAS = control beliefs about stress, CR = cognitive reappraisal, IndEff = indirect effect.

^a^Empirical 95% confidence interval does not overlap with zero.

## Data Availability

The datasets generated during and/or analyzed during the present study are available from the corresponding author on reasonable request.
